# Supplementary material for: Ineffective behavioral rescue despite partial brain Dp427 restoration by AAV9-U7-mediated exon 51 skipping in mdx52 mice
Source: Mol Ther Nucleic Acids. 2025 Nov 19;36(4):102779. doi: 10.1016/j.omtn.2025.102779 (PMC12718193; doi:10.1016/j.omtn.2025.102779)
Supplement: Document S1. Figures S1–S4 [file mmc1.pdf]

OMTN, Volume *36*

Supplemental information

Ineffective behavioral rescue despite partial brain Dp427 restoration by AAV9-U7-mediated exon 51 skipping in *mdx52* mice

Ophélie Vacca, Amel Saoudi, Mathilde Doisy, Xaysongkhame Phongsavanh, Olivier Le Coz, Cathy Nagy, Julia Kuzniar, Cyrille Vaillend, and Aurélie Goyenvalle

SUPPLEMENTAL INFORMATION

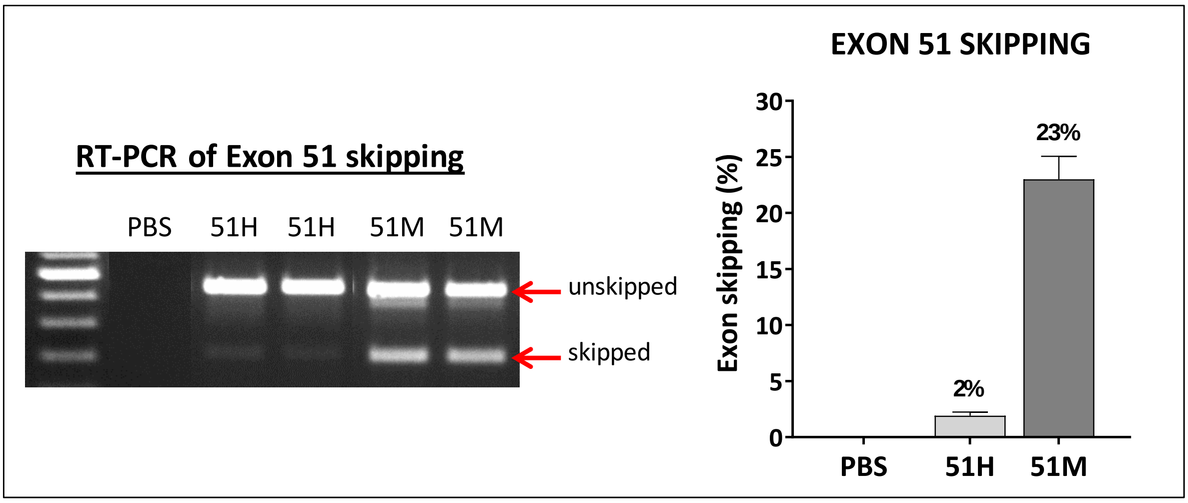

<u>Figure S1</u>: Comparison of exon 51 skipping efficiency using U7-exon51 with human versus murine sequences in *mdx52* mice. (Left panel): RT-PCR analysis of exon 51 skipping after intramuscular injection of scAAV9-U7-51H (human sequence) or scAAV9-U7-51M (murine sequence) in *mdx52* mice at an equivalent dose of 1×10^{12} vg. Lane 1: DNA ladder; Lane 2: PBS-injected *mdx52* mouse (negative control); Lanes 3 & 4: scAAV9-U7-51H-injected *mdx52* mice; Lanes 5 & 6: scAAV9-U7-51M-injected *mdx52* mice. (Right panel): Quantification of exon 51 skipping efficiency as a percentage, based on RT-PCR gel analysis. Data are presented as mean ± SEM and analyzed using Unpaired t test test; ***p < 0.001.

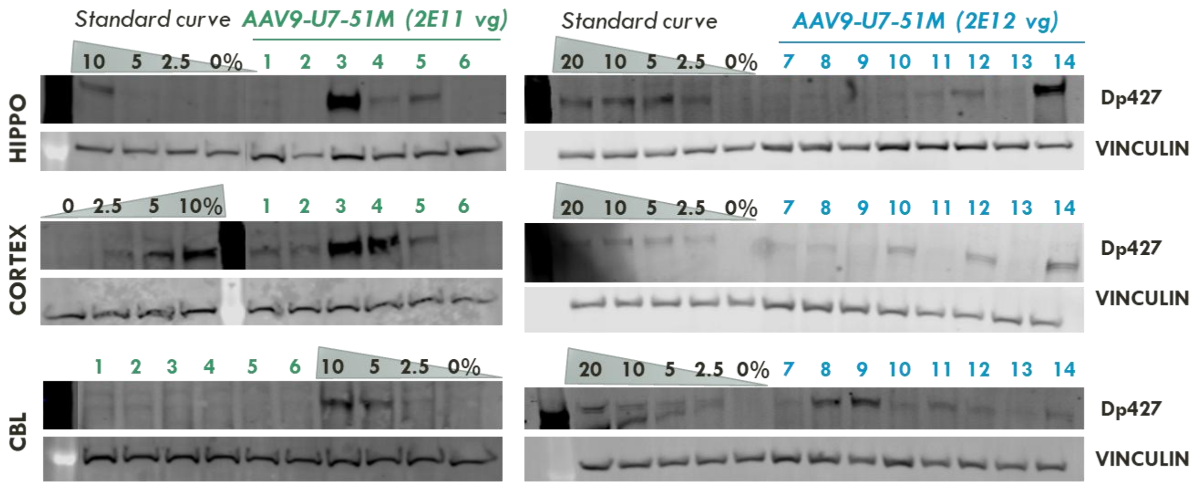

<u>Figure S2</u>: Western blot analysis of Dp427 restoration in *mdx52* mouse brain after AAV9-U7-51M administration. Representative western blot images of Dp427 and vinculin (loading control) in hippocampus (HIPPO), cortex, and cerebellum (CBL) from *mdx52* mice injected intracerebroventricularly with AAV9-U7-51M at two doses: 2E11 vg (mice 1–6, green) and 2E12 vg (mice 7–14, blue). A standard curve was included on each gel, generated by mixing increasing proportions of wild-type (WT) brain extracts (containing Dp427) with decreasing proportions of *mdx52* brain extracts (lacking Dp427) to obtain 20%, 10%, 5%, 2.5%, and 0% of normal Dp427 levels, while maintaining a constant total amount of protein per lane. This approach ensures comparable vinculin levels across the dilution series. Quantification of Dp427 restoration was normalized to vinculin to correct for any minor variation in protein loading. Each western blot was performed in duplicate, and the quantifications reported in the manuscript correspond to the mean of duplicate experiments for each animal. The linear regressions of the standard curves consistently showed coefficients of determination (R^2) ≥ 0.89, confirming the reliability and linearity of Dp427 quantification. Restoration of Dp427 was variable across animals and brain regions.

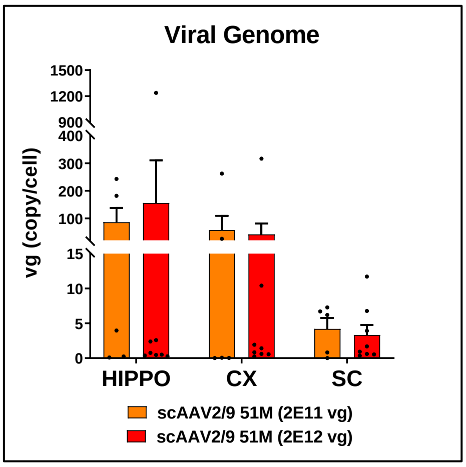

<u>Figure S3</u>: Biodistribution of the scAAV-U7-51M vector. Quantification of the viral genome copies via qPCR on genomic DNA in the HIP, CX and SC, 9 weeks after scAAV-U7-51M ICV injection, comparing low- and high-dose treatments (n=7 for the low-dose in orange; n=8 for the high dose in red).

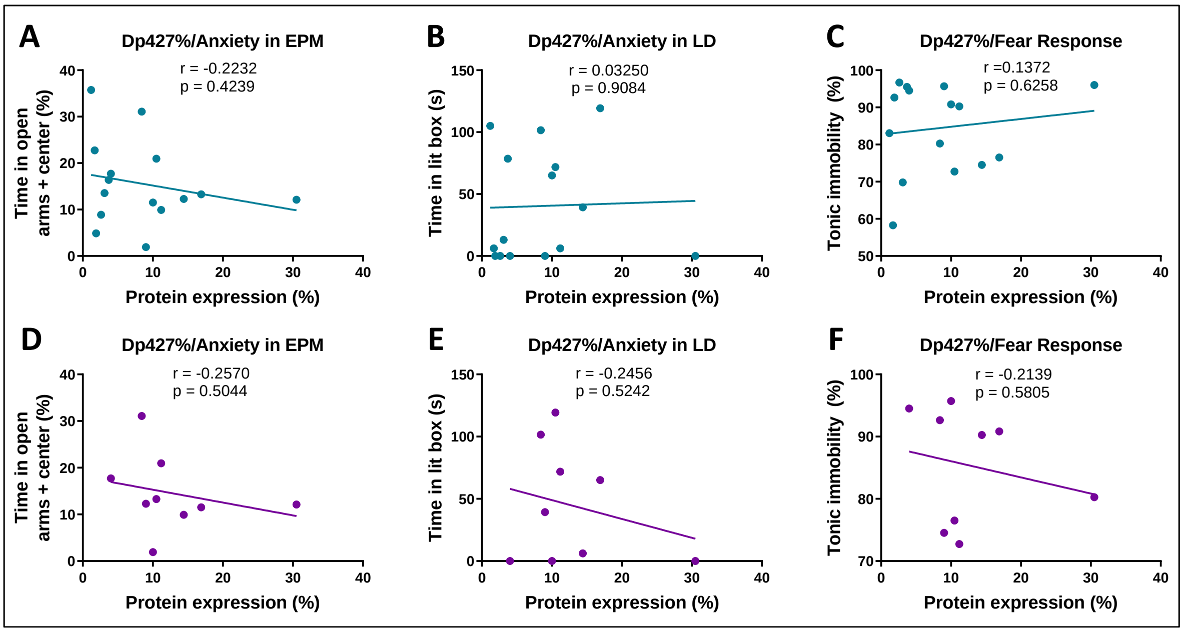

Figure S4: Correlation of Dp427 Restoration with Anxiety- and Fear-Related Behaviors. This figure displays the correlation between the percentage of Dp427 restoration and behavioral measures of anxiety and fear in treated *mdx52* mice. Panels A–C show correlations for all mice (n=15) in the study, while panels D–F present correlations for the post-hoc selected subset of mice (n=9). Specific behavioral parameters are: time spent in open arms and in the center in the elevated plus maze (EPM) (A, D), time spent in the lit box in the light-dark choice test (LD) (B, E), and tonic immobility in the unconditioned fear response test (C, F). Pearson correlation coefficients (r), P values (p) and linear regression lines are shown.
